# Antisense oligonucleotide‐based α‐synuclein targeting as a disease‐modifying treatment for Dementia with Lewy bodies (DLB) disease

**DOI:** 10.1002/alz.091791

**Published:** 2025-01-09

**Authors:** Rijwan Uddin Ahammad, Brian Spencer, Andre D. G. Leitao, Robert A. Rissman

**Affiliations:** ^1^ Department of Neurosciences, University of California San Diego, La Jolla, CA USA; ^2^ Alzheimer’s Therapeutic Research Institute, Keck School of Medicine, University of Southern California, San Diego, CA USA

## Abstract

**Background:**

Dementia with Lewy bodies (DLB) is characterized by the accumulation of α‐synuclein (α‐syn) as well as Alzheimer’s disease (AD) pathology, which includes the accumulation of amyloid beta (Aß) in plaques and phosphorylated tau in tangles, leading to neurodegeneration, cognitive loss and dementia. In DLB and other synucleinopathies, α‐syn oligomers and proto‐fibrils are thought to be mechanistically linked to the pathogenic neurodegenerative process. AD and related disorders (ADRD) are leading causes of dementia in the aging population and although new approaches are being tested, to date no disease‐modifying therapies are available.

**Method:**

We utilized 2’‐OMe modified ASO‐syn or ASO‐scrambled, conjugated with ApoB^11^, which can cross the blood‐brain barrier. ApoB^11^:2’‐OMe ASO peptides were systematically delivered to DLB tg/non‐tg mice to evaluated pharmacokinetics, pharmacodynamics and toxicology profile and determine its effects on α‐syn and neurodegeneration using qPCR, immunohistochemistry and Western blot.

**Result:**

We found that ApoB^11^:2’‐OMe ASO targeted to α‐syn was well tolerated and safe in DLB tg mice without evidence of toxicity and it was successfully delivered to the mice brain. In addition, treatment with ApoB^11^:2’‐OMe ASO targeted to α‐syn significantly reduced α‐syn aggregates in brains of DLB tg mice. Our ongoing experiment shows changes in neuronal survival, motor performance and cognition with the treatment.

**Conclusion:**

Collectively, our findings suggest that the reduction of α‐syn through the use of systemically‐delivered ApoB^11^:ASO α‐syn is a safe disease‐modifying treatment for DLB and may hold promise as a future therapeutic for other synucleinopathies.

